# Low-Dose Acyclovir Reduces the Reactivation of Herpes Simplex and Varicella Zoster Viruses After Allogeneic Stem Cell Transplantation

**DOI:** 10.7759/cureus.75270

**Published:** 2024-12-07

**Authors:** Sorana G Ursu, Mohammad M Alhousani, Gina Patrus, Salman Fazal

**Affiliations:** 1 Division of Hematology and Cellular Therapy, Allegheny Health Network Cancer Institute, Pittsburgh, USA; 2 Division of Hematology and Oncology, Allegheny Health Network Cancer Institute, Pittsburgh, USA

**Keywords:** acyclovir, allogeneic stem cell transplantation, herpes simplex virus, varicella zoster virus, viral reactivation

## Abstract

Introduction: Reactivation of herpes simplex virus (HSV) and varicella zoster virus (VZV) is a potential complication following allogeneic stem cell transplantation (alloSCT). Since different doses and durations of acyclovir prophylaxis may be utilized across transplant centers, this study aimed to evaluate the effectiveness of a lower dose of acyclovir in preventing HSV and VZV reactivation in alloSCT recipients within our institution.

Methods: A retrospective chart review was conducted for patients who underwent alloSCT between April 2016 and May 2023. Patients received acyclovir 400 mg orally twice daily as prophylaxis, starting before stem cell infusion and continuing for two years after alloSCT, with ongoing use while on immunosuppressive therapies. Outcomes measured include incidence of HSV and VZV reactivation, timing of viral reactivation, acyclovir-related adverse events, and hospitalizations due to viral reactivation. Pertinent data collected were risk factors for viral reactivation in patients such as immunosuppressive therapies and chemotherapy regimens. Descriptive statistics were used for data analysis.

Results: A total of 246 patients were included in this study. The majority of patients were male with a median age of 60 years (range 20-76) and a diagnosis of acute myeloid leukemia. HSV reactivation occurred in 10 out of the 246 patients (4%) and none had VZV reactivation. The median time to HSV reactivation was 100 days (range 10-1400). No patient had acyclovir-related adverse events such as acute kidney injury, neurotoxicity, or rash. Hospitalization related to HSV reactivation occurred in four of the 10 affected patients (40%).

Conclusions: Oral acyclovir 400 mg taken twice daily for prophylaxis in alloSCT recipients is both effective and well-tolerated. This lower-dose regimen effectively prevents HSV and VZV reactivation without compromising efficacy or patient safety.

## Introduction

Viral reactivation is a significant cause of morbidity and mortality in patients following allogeneic stem cell transplantation (alloSCT). Among the most clinically relevant reactivations are those of herpes simplex virus (HSV) types 1 and 2, and varicella zoster virus (VZV), because viral reactivation rather than a primary infection is the etiology for most who develop HSV or VZV infection following alloSCT [1]. HSV reactivation occurs early during pre-engraftment shortly after initiation of immunosuppressive therapy (typically 2-3 weeks post-alloSCT) [2]. Similarly, the incidence of symptomatic VZV reactivation ranges from 13% to 55% within the first year following stem cell transplantation with a median of five months (range two to 10 months) [3,4].

Without antiviral prophylaxis, HSV disease occurs in 70-80% of alloSCT recipients if they are seropositive. With the initiation of acyclovir prophylaxis, the incidence of the disease has fallen to less than 5% and has shifted to a later time following alloSCT as compared to patients who have not received prophylaxis (median time after alloSCT 78 days vs 9 days; p=0.006) [5]. VZV reactivation rates, on the other hand, have been reported to be 20-45% during the first year after the transplant in the absence of antiviral prophylaxis, highlighting the importance of preventive strategies [6-8].

Many risk factors have been linked to the development of HSV or VZV reactivation amongst alloSCT recipients. The type of donor used and the intensity of the conditioning regimen are important in the setting of HSV reactivation. For VZV reactivation, age, pre-transplant diagnosis of leukemia and other lymphoproliferative disorders, cluster of differentiation (CD)34+ cell-selected peripheral blood stem cells, and development of acute or chronic graft-versus-host-disease (GVHD) are the associated risk factors [9-11].

Acyclovir prophylaxis is recommended for the first year post-transplant for VZV-seropositive autologous and allogeneic recipients [12]. For HSV prophylaxis, a dose range of acyclovir 400 mg to 800 mg twice daily is recommended and covers early viral reactivation. However, for late HSV reactivation and for VZV prophylaxis, the dose of acyclovir 800 mg twice daily is required for adequate prophylaxis [12]. According to the results of two randomized studies, the recommended oral dose of acyclovir is 800 mg twice daily reported less than 5% reactivation in the first year after stem cell transplant [7,13]. Many transplant centers across the nation have challenged this approach given the high risk for nephrotoxicity with concurrent use of antimicrobials or immunosuppressive medications in alloSCT patients [14].

We aimed to evaluate the impact of using a lower dose of oral acyclovir (400 mg twice daily) on the incidence of breakthrough of HSV and VZV reactivation in alloSCT recipients.

## Materials and methods

This retrospective cohort study included adult patients aged 18 to 89 years old who underwent alloSCT at Allegheny Health Network Cancer Institute between April 2016 and May 2023. All patients received oral acyclovir 400 mg twice daily, starting prior to stem cell infusion and continuing for two years after alloSCT, with ongoing use while on immunosuppression as per institutional guidelines. Exclusion criteria included pregnant or incarcerated patients and patients who had undergone autologous stem cell transplantation. The primary objective of this single-center study was to evaluate the effect of a lower dose of acyclovir on the incidence of HSV and VZV reactivation. Secondary objectives included the timing of viral reactivation, acyclovir-related adverse events (acute kidney injury, neurotoxicity, and rash), and hospitalizations due to viral reactivation. Risk factors for viral reactivation, including immunosuppressive therapies for treating GVHD and the chemotherapy conditioning regimen, were also collected. HSV and VZV reactivation was defined as a culture-positive lesion. Acute GVHD was graded according to the Glucksberg classification and chronic GVHD was graded according to the National Institute of Health Consensus Criteria [15,16]. The Allegheny Health Network Institutional Review Board approved this study (approval number 000015120).

Statistics

Patient demographic and characteristic data were analyzed by frequency (percentage) for categorical variables and mean (standard deviation) or median (interquartile range) for continuous variables, as appropriate.

## Results

Patient demographics

A total of 246 patients were included in this study. The majority of patients were males, with a median age of 60 years (range 20-76) and diagnosed with acute myeloid leukemia. Of the cohort, 164/246 (67%) of patients received reduced-intensity conditioning chemotherapy prior to alloSCT and 162/246 (66%) of patients received an unrelated donor transplant. Baseline patient demographic characteristics are depicted in Table 1. 

**Table 1:** Baseline characteristics of the study cohort (n=246) AML, Acute myeloid leukemia; MDS/MPD, Myelodysplastic syndrome/Myeloproliferative disorder; ALL, Acute lymphoblastic leukemia *Only age is depicted in years; **Other types of diagnoses include chronic myelogenous leukemia (n=4), chronic lymphocytic leukemia (n=1), multiple myeloma (n=2), severe aplastic anemia (n=9), and other leukemias (n=2)

Characteristics	Number of patients (%)*
Age, years – median (range)	60 (20-76)
Sex	
Male	159 (65)
Female	87 (35)
Diagnosis	
AML	126 (51)
MDS/MPD	71 (29)
ALL	21 (9)
Lymphoma	10 (4)
Other^**^	18 (7)
Donor type	
Related	84 (34)
Unrelated	162 (66)
Conditioning regimen	
Myeloablative	82 (33)
Reduced intensity	164 (67)

Outcomes

HSV reactivation occurred in 10/246 (4%) of patients, while no cases of VZV reactivation were observed. Among the 10 patients who experienced HSV reactivation, two patients had multiple episodes (Table 2). One patient experienced four HSV reactivation events. At the time of the first HSV reactivation, this patient had acute grade three gastrointestinal (GI) and skin GVHD. During subsequent reactivations, the patient developed severe chronic GVHD of the liver and the mouth. Risk factors at the time of HSV reactivation were administration of thymoglobulin as a part of GVHD prophylaxis and GVHD treatment with immunosuppressive therapies (prednisone, ruxolitinib, and tacrolimus).

The second patient had two episodes of HSV reactivation. This patient had acute grade three GI GVHD at the time of the the first HSV reactivation. Risk factors at the time of HSV reactivation included thymoglobulin administration as part of GVHD prophylaxis, a myeloablative conditioning chemotherapy regimen, and GVHD treatment with immunosuppressive therapies (beclomethasone, budesonide, prednisone, ruxolitinib, and tacrolimus) (Table 2).

**Table 2:** Patients in HSV reactivation group FB, Fludarabine, busulfan; FBTBI, Fludarabine, busulfan, and total body irradiation; FLUMELCY, Fludarabine, melphalan, and cyclophosphamide; Thymo, thymoglobulin; CY, Cyclophosphamide; GI, Gastrointestinal; HSV, Herpes simplex virus

Patient Number	HSV source	Conditioning regimen	GVHD at the time of HSV reactivation	Immunosuppressant at the time of HSV reactivation	Time of HSV reactivation after transplant (days)
1	Genital	FB2/thymo	Severe liver and mouth	Tacrolimus, prednisone, ruxolitinib	523
1	Genital	FB2/thymo	Severe liver and mouth	Tacrolimus, prednisone	1400
1	Nasopharyngeal	FB2/thymo	None	Tacrolimus, ruxolitinib, prednisone	116
1	Skin	FB2/thymo	Grade 3 GI and skin	Tacrolimus, ruxolitinib, prednisone	126
2	Mouth	FBTBI/thymo	None	Tacrolimus, ruxolitinib, prednisone, budsonise	427
2	Throat	FBTBI/thymo	Grade 3 GI	Tacrolimus, budesonide, beclomethasone, ruxolitinib, prednisone	23
3	Throat	FBTBI/thymo	Grade 3 GI	Tacrolimus, budesonide, beclomethasone, ruxolitinib, prednisone	56
4	Throat	FBTBI/thymo	Grade 3 GI	Tacrolimus, budesonide, beclomethasone, prednisone	83
5	Throat	FLUMELCY	None	Sirolimus, mycophenolate	461
6	Genital	FB2/thymo	None	None	38
7	Genital	FB2/thymo	None	Tacrolimus, topical triamcinolone	76
8	Mouth	FB2/thymo	None	Tacrolimus, prednisone	128
9	Throat	FBTBI/CY	None	None	25
10	Throat	FB2/CY	None	Tacrolimus, mycophenolate	10

The most common sites of HSV reactivation were the throat (6/14, 43%), the genital region (4/14, 29%) and the mouth (2/14, 14%). At the time of HSV reactivation, four out of 10 patients (40%) had any grade GVHD and eight out of 10 patients (80%) received immunosuppressive therapies. The most commonly administered immunosuppressive therapies were tacrolimus followed by prednisone. 

The median time to HSV infection was 100 days (range 10-1400). No patients documented acyclovir-related adverse events such as acute kidney injury, neurotoxicity, or rash. Hospitalization due to viral reactivation occurred in four out of 10 patients (40%) of patients.

## Discussion

This retrospective cohort study evaluated the incidence of HSV and VZV reactivation in alloSCT recipients receiving a lower prophylactic dose of oral acyclovir at 400 mg twice daily. Our findings showed that the incidence of HSV reactivation was 4%, with no cases of VZV reactivation. The median time to HSV reactivation was 100 days (range 10-1400 days), and no patients experienced acyclovir-related adverse events. Hospitalization due to viral reactivation occurred in four out of the 10 affected patients (40%).

Our institutional practice of using a lower dose of oral acyclovir (400 mg twice daily) prompted a comparison with the existing literature on acyclovir dosing regimens in alloSCT recipients. The literature revealed a considerable variation in acyclovir dosing, with daily doses ranging from 200 mg to 3200 mg, and differing definitions of HSV and VZV reactivation.

Mascarenhas et al. reported an incidence of VZV infection (categorized as dermatomal, involvement of one to two dermatomes, or disseminated involvement of greater than two dermatomes or extra-cutaneous involvement) of 2.8% at one year and 5.8% at two years after transplant in alloSCT recipients treated with oral acyclovir 400 mg twice daily [14]. In contrast, Kawamura et al. used a lower oral acyclovir dose (200 mg daily) and found a cumulative incidence of 3.6% for HSV disease [17]. Fei et al. reported no instances of VZV reactivation in autologous stem cell transplant recipients on oral acyclovir 400 mg twice daily or oral valacyclovir 500 mg daily [18]. Dennison et al. used oral acyclovir 200 mg daily or 5 mg/kg/day if body weight was less than 40 kg for one year after transplant and reported a 2.5% incidence of herpes zoster in their cohort [19]. Seo et al. conducted a meta-analysis of six studies involving alloSCT recipients (n=3420), and showed an overall 7.8% incidence of herpes zoster in the prophylaxis group versus 25.6% in the control group, with a pooled relative risk of 0.31 (95% CI, 0.26-0.37). The acyclovir dose ranged from 200 mg/day up to 3200 mg/day [20]. 

In comparison to these studies, our findings indicated a 4% incidence of HSV reactivation, which is slightly lower than that observed in randomized controlled trials (5%) using higher oral acyclovir doses (800 mg twice daily) [9]. Additionally, our study showed 0% VZV reactivation, which is lower than the 5% VZV-only disease reported by Boeckh et al. during the first year post-transplant using oral acyclovir 800 mg twice daily [14]. Our results are comparable to those from studies using oral acyclovir 400 mg twice daily, where the incidence of VZV reactivation ranged from 0% to 3.6% [14,17,18]

The Guidelines for Preventing Infectious Complications among Hematopoietic Cell Transplant Recipients recommend oral acyclovir doses ranging from 400 to 800 mg twice daily to prevent early reactivation of HSV among seropositive hematopoietic stem cell transplant recipients. The guidelines specify using oral acyclovir 800 mg twice daily to prevent late reactivation of HSV. For prophylaxis against VZV reactivation, oral acyclovir 800 mg twice daily for a year is recommended [12]. Our institutional practice of using a lower dose of oral acyclovir (400 mg twice daily) had a median time to HSV reactivation of 100 days post-transplant, which falls between the threshold of early (within 100 days) and late reactivation (after 100 days). Of the 10 patients who experienced HSV reactivation, seven (70%) had early reactivation, suggesting that the lower dose of acyclovir was effective in preventing early HSV reactivation, as compared to the guidelines. Late reactivation occurred in three patients (30%) which may justify the use of the higher dose (oral acyclovir 800 mg twice daily) in these cases.

Additionally, for long-term prophylaxis, the higher dose of acyclovir is recommended for maximal viral suppression and minimization of resistance. For seropositive alloSCT recipients, acyclovir 800 mg twice daily is recommended for one year post-transplant [12]. Our institutional standard is to use acyclovir prophylaxis for two years in alloSCT recipients. Extended duration of acyclovir may be required based on identified risk factors for HSV and VZV reactivation, such as continued use of immunosuppressive therapy for GVHD. 

Several risk factors for HSV reactivation have been identified, including the use of myeloablative conditioning regimens and the intensity and duration of immunosuppressive. In our cohort, five out of the 10 patients with HSV reactivation (50%) received a myeloablative conditioning chemotherapy regimen and seven (70%) received thymoglobulin (Table 2).

Our institutional standard for GVHD prophylaxis is tacrolimus, mycophenolate mofetil, and post-transplant cyclophosphamide for haploidentical donors. Patients are switched from tacrolimus to sirolimus if the former is not tolerated. At the time of HSV reactivation, four out of the 10 affected patients (40%) had any grade of GVHD, five (50%) were receiving prednisone, seven (70%) were on tacrolimus and one patient was on sirolimus. In our institution, prednisone or methylprednisolone one to two mg/kg/day is started for treatment of acute GVHD, depending on grade and organ involvement. Ruxolitinib is indicated for treatment of steroid-refractory acute GVHD in adult and pediatric patients (12 years and older) [21]. Ruxolitinib was used for steroid-refractory acute GVHD in three out of 10 patients (30%) who exhibited HSV reactivation. This use of ruxolitinib aligns with the current literature, where 35-50% of acute GVHD cases become refractory to steroids, and ruxolitinib is increasingly used in such cases [22].

Limitations

There are several limitations to this study. First, its retrospective design is susceptible to biases and we only included confirmed HSV and VZV reactivations based on positive cultures. It is possible that additional patients experienced suspected (not confirmed) HSV or VZV reactivations that were treated with antiviral therapy. Additionally, there could have been missing documentation in the medical records of ICD-10 diagnosis codes of confirmed or suspected HSV or VZV reactivations and these patients were missed. Given the labor-intensive nature of reviewing medical records over a seven-year period, we relied on an electronic report to capture culture-positive lesions of HSV or VZV of any patient seen by a provider within our clinic or admitted to the hospital. We then manually reviewed the electronic report and excluded autologous stem cell transplant recipients and patients with hematologic malignancies who had not undergone alloSCT, as their acyclovir dosing regimens differed from those of alloSCT recipients. This could have introduced a confounding variable [23].

## Conclusions

Our study demonstrated that a lower dose of oral acyclovir (400 mg twice daily) was effective in preventing HSV and VZV reactivation in alloSCT recipients without compromising safety or efficacy. This dosing regimen is well-tolerated, reduces patient pill burden, and minimizes potential side effects, offering a practical alternative to higher doses. Furthermore, by maintaining therapeutic efficacy while lowering the total daily dose, this approach may enhance adherence to antiviral prophylaxis in alloSCT recipients, who often face complex medication regimens. Additionally, our findings suggest that the lower acyclovir dose may be particularly advantageous in reducing the risk of acyclovir-associated renal toxicity and other adverse effects, making it a safer option for this vulnerable patient population. Long-term use of acyclovir prophylaxis after a transplant may be necessary for certain patients on continuous immunosuppressive therapy, highlighting the importance of a favorable adverse event profile. These results support the use of a reduced-dose acyclovir regimen as an effective, safe, and more patient-friendly strategy for preventing HSV and VZV reactivation in alloSCT recipients.
